# Time-varying pattern of recurrence risk for localized melanoma in China

**DOI:** 10.1186/s12957-019-1775-5

**Published:** 2020-01-04

**Authors:** Xizhi Wen, Dandan Li, Jingjing Zhao, Jingjing Li, Tao Yang, Ya Ding, Ruiqing Peng, Baoyan Zhu, Fuxue Huang, Xiaoshi Zhang

**Affiliations:** 1 Biotherapy Center, State Key Laboratory of Oncology in South China, Collaborative Innovation Center for Cancer Medicine, Sun Yat-sen University Cancer Center, Guangzhou, 510060 Guangdong People’s Republic of China; 2 Department of Orthopedics, Guangdong Provincial People’s Hospital, Guangdong Academy of Medical Sciences, Guangzhou, 510060 Guangdong People’s Republic of China

**Keywords:** Melanoma, Recurrence hazard, Chinese population

## Abstract

**Background:**

Acral and mucosal melanomas are rarely seen in Caucasians but common in China. There are limited data on the recurrence characteristics for these patients. This study aimed to identify the recurrence pattern for localized melanoma in China, especially acral and mucosal subtypes.

**Methods:**

Patients with localized melanoma who underwent radical resection between January 1999 and December 2014 in southern China were retrospectively reviewed. Survival and annual recurrence hazard were analyzed by Kaplan–Meier method and hazard function, respectively.

**Results:**

Totally, 1012 patients were included (acral melanoma 400; chronic sun-induced damage (CSD)/non-CSD melanoma 314; mucosal melanoma 298). Recurrence was recorded in 808 patients (localized 14.1%; regional 29.6%, and distant 56.3%). Mucosal melanoma had local and M1c stage recurrence more frequently than cutaneous melanoma, but less frequent regional node relapse. There was no difference in recurrent site distribution between acral and CSD/non-CSD melanoma. The annual recurrence hazard curve for the entire cohort showed a double-peaked pattern with the first major peak in the second year after surgery and the second peak near the seventh year. Mucosal melanoma had a higher recurrence risk than cutaneous melanoma. Acral melanoma had a lower flat recurrence peak than CSD/non-CSD melanoma. Tumor thickness > 4.0 mm, ulceration, positive regional nodes, and wound infection were associated with a higher recurrence risk in cutaneous melanoma. Adjuvant therapy reduced the recurrence risk of cutaneous melanoma but not of mucosal melanoma.

**Conclusions:**

This is a large cohort about the rule of recurrence risk in acral and mucosal melanoma and will provide an initial framework for development of surveillance and adjuvant strategy for Chinese melanoma patients.

## Introduction

Malignant melanoma is a heterogenous group of melanocytic neoplasms diagnosed in approximately 20,000 people annually in China [1]. Most patients diagnosed with localized disease are considered curable. However, the risk of loco-regional recurrence or distant metastasis remains high, at between 30 and 60% in cutaneous melanoma and between 59 and 100% in mucosal melanoma [2, 3]. Therefore, understanding the regularity of recurrence is essential for guiding appropriate treatment in localized melanoma patients.

There are significant demographic and ethnic differences in melanoma. In the Chinese population, melanoma arising from the skin accounts for between 50 and 70%, with the acral areas being the most common primary sites. Mucosal melanoma is the second most common subtype, with a percentage incidence of between 22 and 25% [4]. For Caucasians, approximately 90% of melanoma arise from the skin, commonly on skin of non-acral sites, while melanoma arising from the mucous membranes and acral areas account for between 1 and 5% [5]. Acral and mucosal melanomas have been reported to have distinct genetic and clinical characteristics and a poorer prognosis [6–9]. The existing melanoma follow-up schedules in China are based on evidence derived from western countries. Postoperative recurrence characteristics of localized melanoma in Chinese patients, especially regarding the acral and mucosal types, are scarce.

In most studies about melanoma, the recurrence risk is analyzed by survival curves but not hazard rates. Compared with survival curves, the hazard rate can reflect both the magnitude of the recurrence rate and its variation with time. The objective of this study was to identify the patterns and high-risk periods of recurrence in Chinese melanoma patients using the hazard function, especially concerning the acral and mucosal melanoma types, and to provide a reference for the designing of follow-up schedules and adjuvant therapy.

## Methods

### Patients

All histologically confirmed malignant melanoma patients who had received radical resection and admitted in Sun Yat-sen University Cancer Center (SYSUCC) between January 1999 and December 2014 were retrospectively analyzed. The patients included in this study met the following criteria: (1) histologically confirmed malignant melanoma; (2) no distant metastasis was found before operation; (3) patients underwent radical surgery; and (4) patients were followed up for at least 3 months. Patients were excluded from the study if they received incomplete resection or neoadjuvant therapy or died of surgical complications. Patients were staged or restaged retrospectively according to the American Joint Committee on Cancer (AJCC) staging system (7th edition). Because sentinel lymph node biopsy is not routinely carried out at our center, only some of the stage I and stage II patients in this study received regional lymph node biopsy. The ultrasonography of regional lymph nodes was routinely carried out as an alternative. Regional lymph node dissection was routinely applied for clinical stage III patients.

### Classification of recurrence

Recurrent sites were categorized into five sites corresponding with AJCC staging criteria, namely: (1) local recurrence; (2) regional nodes, including in transit metastasis; (3) distant skin, soft tissue including muscles, and/or nonregional lymph nodes (M1a); (4) the lungs with or without skin or soft tissue involvement (M1b); and (5) other distant sites, including the liver, gastrointestinal, bone, central nervous system, adrenal glands, eye, and any other sites, and death due to metastases (M1c). If multiple recurrences occurred at the same time, recurrences were counted as one event. A site was classified according to the site with the worst expected prognosis based on staging criteria indicating that non-pulmonary visceral metastases represented the poorest prognostic group, followed by pulmonary metastases, distant skin and soft tissue, and local regional recurrence only.

### Statistical analysis

The statistical methods referred to Zhu JF’s study [10]. Recurrence-free survival (RFS) was measured from the time of surgery to the earliest occurrence of relapse (loco-regional or distant), last follow-up, or death from melanoma. Patients who were lost to follow-up or alive at the end of the study were censored for data analysis. RFS was estimated by the Kaplan–Meier method and compared with the log-rank test. For the multivariable analyses, we used two methods to check the proportional hazards (PH) assumption: graphical and time-dependent variable approaches. The variables that did not satisfy PH hypothesis were defined as time-dependent variables, which were introduced into the Cox regression model to form Time-Depended Cox Regression Model. The comparison of frequency between each group was performed using the chi-square test. The kernel smoothing method was used to estimate the annual hazard rates and display the graphical display of RFS. A two-sided probability value of less than 0.05 was considered statistically significant. All the statistical analyses were performed using the Stata statistical software package.

## Results

### Patient characteristics

Total of 1012 patients fulfilling the inclusion criteria were included in this study. The median age was 53 years (range, 14–89 years), and the study comprised 534 males and 478 females. Among 714 patients with cutaneous melanoma, 400 were diagnosed with acral melanoma and 314 were diagnosed with melanoma on the skin with chronic sun-induced damage (CSD) or melanoma on skin without chronic sun-induced damage (non-CSD). There were 298 patients with mucosal melanoma included in this study, accounting for 29.4% of all patients. The most common sites of mucosal melanoma were the head and neck (63.1%), followed by the genitourinary tract (21.5%) and the digestive tract (14.1%). In addition, there were 4 patients (1.3%) with mucosal melanoma originating in the respiratory tract. There were 368 cutaneous melanoma patients and 105 mucosal melanoma patients who received adjuvant therapy postoperatively. The clinicopathological characteristics of these patients are detailed in Table 1.

**Table 1:** Clinical and pathological characteristics of patients with localized melanoma

Characteristics	No.	%
Age (years)		
Median	53	
Range	14-89	
Sex		
Male	534	52.8
Female	478	47.2
Clinical subtypes		
Acral	400	39.5
CSD/non-CSD	314	31.0
Mucosal	298	29.4
Anatomic site for mucosal melanoma		
Head and neck	188	63.1
Genitourinary tract	64	21.5
Digestive tract	42	14.1
Respiratory tract	4	1.3
AJCC stage (for cutaneous melanoma only)		
I	39	5.4
II	361	50.6
III	314	44.0
Ulceration (for cutaneous melanoma only)		
Present	342	47.9
Absent	372	52.1
Primary tumor thickness (for cutaneous melanoma only)		
≤ 4.0 mm	228	31.9
> 4.0 mm	354	49.6
Not available	132	18.5
Regional lymph node metastasis (for cutaneous melanoma only)		
Yes	400	56.0
No	314	44.0
Wound infection after surgery (for cutaneous melanoma only)		
Yes	69	9.7
No	645	90.3
Adjuvant therapy for cutaneous melanoma		
Interferon a-2b	246	34.5
Chemotherapy	119	16.7
Radiotherapy	3	0.4
None	346	48.4
Adjuvant therapy for mucosal melanoma		
Chemotherapy	47	15.8
Interferon a-2b	41	13.8
Radiotherapy	17	5.7
None	193	64.8

*CSD* melanoma on skin with chronic sun-induce damage, *non-CSD* melanoma on skin without chronic sun-induced damage

### Overall recurrence patterns

A total of 808 patients (cutaneous melanoma, *n* = 559; mucosal melanoma, *n* = 249) experienced relapse after a median follow-up time of 60 months. The first recurrence was diagnosed within 2 years in 81.8% of the patients, and late recurrences, diagnosed more than 5 years after surgery, were observed in 4.5% of the patients.

**Fig. 1:**
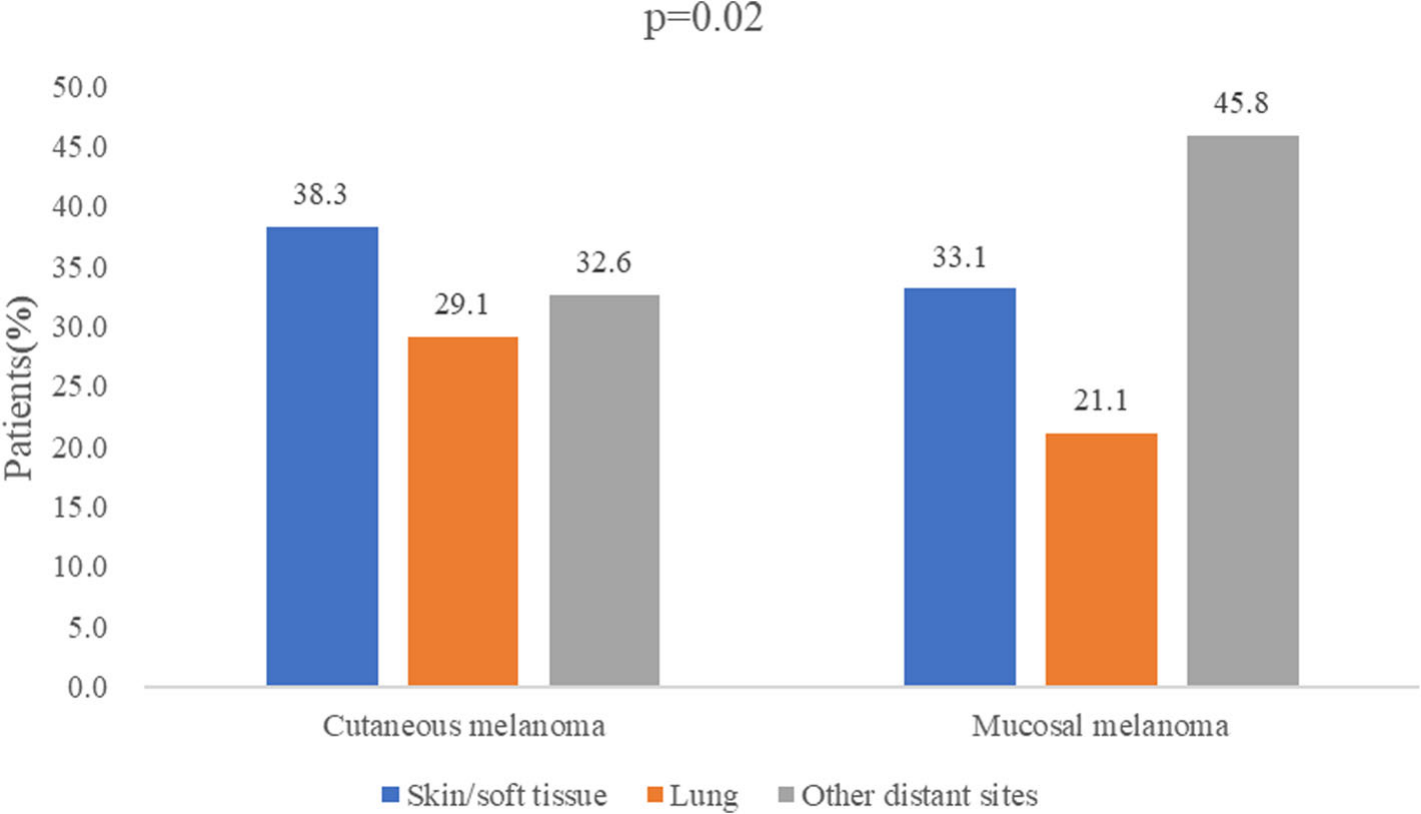
Comparisons of the frequencies of metastatic sites between cutaneous and mucosal melanoma. The most common metastasis site of mucosal melanoma was other distant sites besides the lung, skin, or soft tissue, but for cutaneous melanoma, the most common site was the skin/soft tissue. *P* value refers to the chi-square test of the proportion of the metastasis sites for the two groups

Altogether, 114 (14.1%) of all first recurrences were classified as local, 239 patients (29.6%) presented with regional and 455 (56.3%) with distant metastases. The median times to relapse for local, regional, and distant metastases were 8 months, 12 months, and 10 months, respectively. The distribution of recurrence sites was related to pathological types. Compared to cutaneous melanoma, mucosal melanoma had a significantly higher frequency of local recurrence and a lower frequency of regional recurrence (detailed in Table 2). When the distant recurrence was concerned, there were also differences in the distribution of metastasis sites between cutaneous melanoma and mucosal melanoma. The most common metastasis site of mucosal melanoma was other distant site (M1c), followed by the skin or soft tissue (M1a), and finally the lung (M1b). However, for cutaneous melanoma, the most common site of metastasis was the skin/soft tissue (M1a), followed by other distant sites (M1c), and finally the lung (M1b) (Fig. 1). However, there was no statistical difference in the distribution of the recurrence sites between acral and non-acral cutaneous melanoma (*P* > 0.1). The regional lymph node status at diagnosis correlated with the site of recurrence in cutaneous melanoma. Patients with regional lymph node metastasis at diagnosis were more likely to have distant metastasis. The most common site of recurrence in patients without regional lymph node metastasis at diagnosis was the regional lymph node, followed by distant metastasis (Table 3).

**Table 2:** The relationship between pathological type and the first recurrent sites

Pathology	First recurrent sites		
	Local *n* (%)	Regional	Distant
Cutaneous	33 (5.9%)	213(38.1%)	313(56.0%)
Mucosal	81(32.5%)	26(10.4%)	142(57.0%)
Total	114(14.1%)	239(29.6%)	455(5.3%)

*P* < 0.001

**Table 3:** The relationship between regional lymph node status and the first recurrent sites in cutaneous melanoma

Regional lymph node at diagnosis	First recurrent sites		
	Local	Regional lymph node	Distant metastasis
Not involved	25(8.7%)	142(49.7%)	119(41.6%)
Involved	8(2.9%)	71(26.0%)	194(71.1%)
Total	33(5.9%)	213(38.1%)	313(56.0%)

*P* < 0.005

### Survival analysis according to clinicopathological factors

**Fig. 2:**
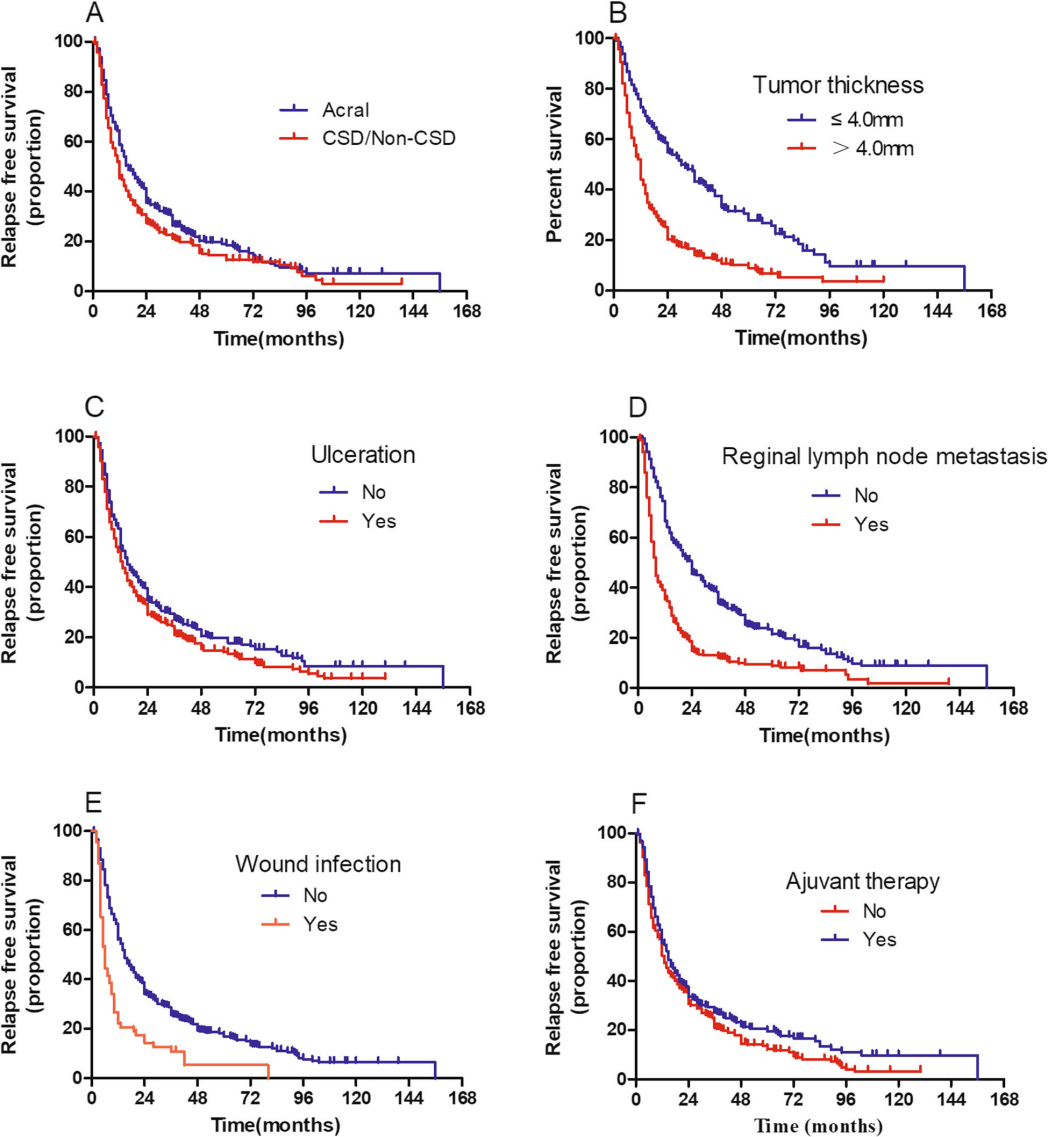
Kaplan–Meier curves for recurrence-free survival (RFS) in 714 patients with cutaneous melanoma analyzed by clinicopathological factors. **a** RFS analysis by primary site; **b** RFS analysis by tumor thickness; **c** RFS analysis by ulceration; **d** RFS analysis by regional lymph node metastasis; **e** RFS analysis by wound infection; and **f** RFS analysis by adjuvant therapy

The median RFS for the whole group was 12.0 months. The life-table survival analysis found that the 1-year, 2-year, 3-year, 5-year, and 10-year recurrence rates for the whole cohort were 38.0%, 28.0%, 26.0%, 17.0%, and 22.0%, respectively. The median RFS for acral and CSD/non-CSD melanoma was 16 months and 12 months, respectively. For cutaneous melanoma, the 1-year, 2-year, 3-year, 5-year, and 10-year recurrence rates were 38%, 26%, 24%, 17%, and 25%, respectively. For mucosal melanoma, the median RFS was 11 months, and the 1-year, 2-year, 3-year, 5-year, and 10-year recurrence rates were 40.0%, 34%, 33.0%, 18.0%, and 0%, respectively. Both univariable and multivariable analyses revealed that primary non-acral type tumors, tumor thickness greater than 4 mm, primary tumor with ulceration, regional lymph node metastasis, and wound infection postoperatively related to a shorter RFS in cutaneous melanoma. Only lymph node metastasis did not satisfy PH hypothesis with its HR value changes with time. The PH assumption test for each variable is listed in Table 4. The Kaplan–Meier survival curve based on each factor is shown in Fig. 2. Adjuvant therapy favored RFS for those with cutaneous melanoma. The anatomic site and postoperative adjuvant therapy, whether chemotherapy, interferon, or radiotherapy, did not relate to the RFS for those with mucosal melanoma. Patient age or sex did not correlate with RFS in the whole cohort. Univariable and multivariable analyses of RFS according to patient’s clinicopathological characteristics are listed in Tables 5 and 6.

**Table 4:** The PH assumption test for variables included in multivariate regression

Time-dependent covariate	Wald *χ*^2^	*P*
Primary site *LN(T_)	3.225	0.073
Tumor thickness *LN(T_)	3.214	0.073
Ulceration *LN(T_)	0.156	0.693
Regipnal lymph node metastasis*LN(T_)	26.93	< 0.01
Wound infection *LN(T_)	3.608	0.057
Adjuvant therapy*LN(T_)	0.061	0.805

**Table 5:** Kaplan–Meier postoperative survival analysis (log-rank test) according to clinical-pathological factors

Variable	mRFS(months)	*P*
Sex		0.135
Male	12	
Female	13	
Age		0.854
≤ 65	13	
> 65	12	
Pathological type		0.001
Mucosal melanoma	11	
Cutaneous melanoma	14	
Clinical subtypes for cutaneous melanoma		0.009
Acral	16	
CSD/N-CSD	12	
Primary site for mucosal melanoma		0.798
Head and neck	11	
Genitourinary tract	12	
Digestive tract	12	
Respiratory tract	13	
Primary tumor thickness for cutaneous melanoma		< 0.01
≤ 4.0 mm	30	
> 4.0 mm	12	
Ulceration for cutaneous melanoma		0.012
Present	13	
Absent	15	
Regional lymph node metastasis for cutaneous melanoma		< 0.001
Yes	8	
No	24	
Stage for cutaneous melanoma		< 0.001
I	82	
II	20	
III	8	
Wound infection for cutaneous melanoma		< 0.001
Yes	6	
No	15	
Adjuvant therapy for cutaneous melanoma		0.021
Yes	15	
No	13	
Adjuvant therapy for mucosal melanoma		0.302
Yes	12	
No	11	

*CSD* melanoma on skin with chronic sun-induce damage, *non-CSD* melanoma on skin without chronic sun-induced damage

**Table 6:** Results of multivariable survival analyses for RFS according to the extended Cox regression model in cutaneous melanoma

Variable	HR	95%CI of HR	*P*
Primary site			< 0.01
CSD/non-CSD	1.46	1.20–1.79	
Acral	1		
Tumor thickness			< 0.01
> 4 mm	1.96	1.59–2.43	
≤ 4 mm	1		
Ulceration			0.005
Present	1.32	1.09–1.61	
Absent	1		
Regional lymph node metastasis			< 0.01
Yes	Exp [1.906–0.519ln(t)]		
No	1		
Wound infection			< 0.001
Yes	2.29	1.70–3.07	
No	1		
Adjuvant therapy			< 0.001
Yes	0.66	0.55–0.80	
No	1		

*CSD* melanoma on skin with chronic sun-induce damage; *non-CSD* melanoma on skin without chronic sun-induce damage; *t* follow-up time after surgery

### Recurrence hazard analysis

**Fig. 3:**
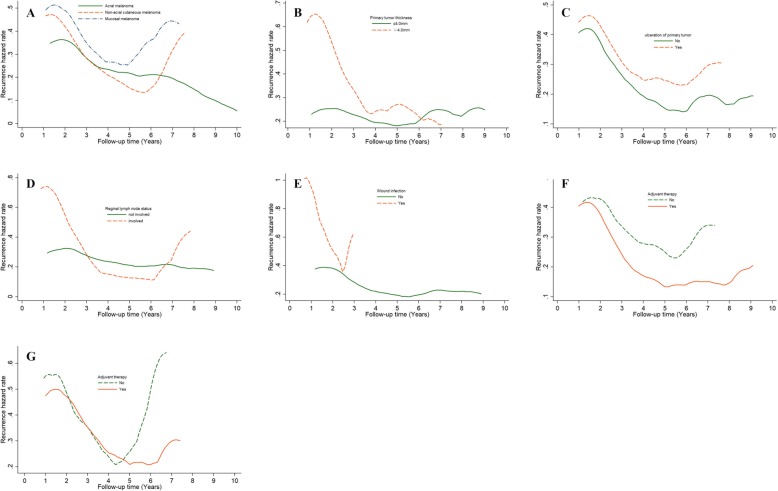
Recurrent hazard curve for the whole cohort

The annual recurrence hazard curve for the entire cohort showed a double-peaked pattern, with a first major recurrence peak at the second year after surgery and covering the first 3 years. The curve then began to fall until the fifth year after surgery, at which point the curves began to rise again, reaching a second peak in the seventh year (Fig. 3).

**Fig. 4:**
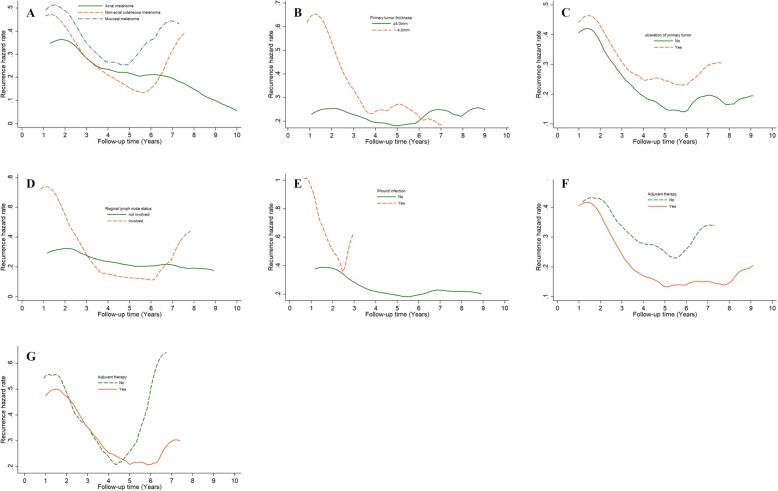
Recurrence hazard analysis according to clinicopathological characteristics. **a** pathological types; **b** primary tumor thickness of cutaneous melanoma; **c**: ulceration of primary lesion in cutaneous melanoma; **d** regional lymph node status for cutaneous melanoma; **e** wound infection of cutaneous melanoma; **f** adjuvant therapy in cutaneous melanoma; **g** adjuvant therapy in mucosal melanoma

Subsequently, we compared the recurrence risk concerning different clinicopathological factors, including primary tumor subtype, tumor thickness, ulceration of primary tumor, regional lymph node status, surgical complications (wound infection), and adjuvant therapy. When the hazard rate was related to primary tumor subtype, the analysis showed that mucosal melanoma had a higher recurrence risk than acral or non-acral cutaneous melanoma throughout the observation period. Acral cutaneous melanoma had a lower recurrence risk than non-acral cutaneous melanoma during the first 3 years after surgery and then rose to show a higher recurrence risk in the following 3 to 7 follow-up years and returned to a steady decline afterwards (Fig. 4a).

In the cutaneous melanoma subgroup, the time distribution of the recurrence risk varied with tumor thickness, ulceration, regional lymph node status, and postoperative complications. Patients with primary tumor thickness greater than 4 mm (T4) demonstrated a more pronounced and variable pattern compared with those with a tumor thickness of ≤ 4 mm (T1-3). Compared with T4 patients, recurrence peaks for T1-3 patients were relatively flat and late (Fig. 4b). The double-peaked pattern was revealed in both ulcerated and non-ulcerated subgroups with the smoothed hazard plots parallel to each other. The annual recurrence hazard curve showed that patients with ulcerative tumors had more recurrences during all analysis periods (Fig. 4c). Patients with regional lymph node metastasis at diagnosis revealed taller and steep peaks than patients with negative regional lymph nodes. The hazard curves crossed during the third to the seventh year after surgery (Fig. 4d). Patients who had wound infection after surgery had a markedly higher and earlier recurrence than those without postoperative wound infection (Fig. 4e).

Adjuvant therapy has varying effects on the recurrence curve in different melanoma subtypes. In cutaneous melanoma, the hazard rates for patients receiving adjuvant therapy was lower with the second peak appeared later and flatter than in the non-adjuvant therapy patients (Fig. 4f). In the mucosal melanoma group, adjuvant therapy reduced the magnitude of the two recurrence peaks, with the peaks remaining relatively stable. The hazard curves crossed during the low recurrence risk period (Fig. 4g).

## Discussion

There have been few reports on the time distribution of recurrence hazard rates for acral and mucosal melanomas. In the present study, we compared recurrence patterns between different melanoma subtypes and demonstrated the presence of two peaks for recurrence risk among Chinese patients with malignant melanoma, most of whom had acral and mucosal subtypes after complete resection. This pattern has been similarly observed in various tumors such as breast cancer, lung cancer, and gastric cancer [10–12].

The site of recurrence has been reported to be a factor influencing survival after relapse. In our study, we demonstrated that most patients recurred as distant metastases, rather than local or regional recurrences, for both cutaneous and mucosal melanoma, which is similar to findings in another report [13]. In subgroup analysis, we found that mucosal melanoma had a higher frequency of local failure than cutaneous melanoma. This pattern indicated that, aside from systemic adjuvant therapy, local therapy such as radiation therapy [14, 15], is also very important for mucosal melanoma. Concerning the pattern of metastatic disease, mucosal melanoma is more likely to metastasize to distant sites other than the skin or the lung, which may explain their short overall survival.

Cutaneous melanoma with regional lymph node involvement at diagnosis had a higher frequency of distant recurrence in our study. Systemic adjuvant therapy should be recommended in preference to local treatment for regional lymph node-positive disease, due to the recurrence pattern of this disease. Patients with negative regional lymph node involvement at diagnosis showed a higher frequency of local regional recurrence than those with positive regional nodes (49.7% vs 26.0%). The possible reason for this difference is that sentinel lymph node biopsy has not been routinely implemented in our center, which may have resulted in not always detecting clinical occult regional lymph node metastasis. Sentinel lymph node biopsy cannot prolong overall survival [16]. Immediate completion lymph node dissection after positive sentinel lymph node biopsy did not increase melanoma-specific survival but was associated with an increased risk of complications such as wound dehiscence or infection, hematoma, and lymphedema [17]. Therefore, in China, where sentinel lymph node biopsy is not widely promoted, it may be a reasonable option to design an intensified follow-up schedule for regional lymph nodes after resection of the primary lesion for patients with clinically negative regional lymph nodes. On the other hand, we found that patients with wound infection after surgery had a higher recurrence risk and a shorter RFS than those without infection, which suggests that a wider range of surgery needs to be considered carefully.

Immune checkpoint inhibitors have had significant success in treating metastatic melanoma, allowing patients to achieve a long-term survival [18]. Patients with lower tumor burden and good performance are usually associated with good response rates and longer survival [19]. A structured surveillance program should have a favorable effect on patient prognosis through detecting a recurrence before a patient becomes symptomatic to ensure early treatment. In contrast to melanoma in Caucasians, melanoma in people of Chinese ethnicity manifests as a higher ratio of acral and mucosal types. However, limited data are available regarding the optimal surveillance strategy for patients diagnosed with mucosal or acral melanomas. The existing follow-up schedules for melanoma in the Chinese population are based on previous experiences with Caucasians. Using hazard rate analyses, we found that the risk of initial recurrence in Chinese melanoma patients tended to peak within the first 1 to 2 years after surgery and covered the first 3 years, which is consistent with another report concerning about Caucasians [20]. Our study results support the NCCN guideline recommendation for a routine follow-up every 3 to 6 months for 3 years, and then every 4 to 12 months for two further years [21]. However, it is not recommended to extend the follow-up interval after 5 years, because of the late peak in the seventh year. Additionally, the first recurrent peak involving the first 3 years found in our study supports a long course of adjuvant therapy.

High-risk factors, including ulceration, tumor thickness, and regional lymph node metastasis, are prognostic factors associated with increased risks of recurrence and a shorter survival time among cutaneous melanoma patients [22]. In this study, a double-peaked pattern was observed in a variety of melanoma subgroups. However, for patients with high-risk factors, they exhibited earlier and higher peaks. These factors should be taken into consideration when considering adjuvant therapy and surveillance strategy.

Acral melanoma has been reported to have a worse prognosis than other types of cutaneous melanoma [6, 23]. Acral melanoma might have a later stage presentation at diagnosis as compared to cutaneous superficial spread melanoma in endemic regions, which may be one of the reasons for its poor prognosis. There are also studies showing that acral melanoma does not differ in terms of patient survival and biological behavior from non-acral melanoma [24]. In the current study, we found that acral melanoma showed a longer RFS and a lower recurrence risk than non-acral cutaneous melanoma. One possible explanation for this was that the stage distribution in our series was uneven. There was a lower proportion of stage III disease included in the acral melanoma group in this study (49.4% for acral melanoma vs 39.8% for non-acral cutaneous melanoma). Another explanation was that acral melanoma was reported to have a significantly lower proportion of BRAF mutations than non-acral cutaneous melanoma [25], and BRAF mutation in melanoma was associated with poor prognosis [26], which may lead to a better prognosis for acral melanoma in this study.

## Conclusions

This study involved a large cohort of Chinese melanoma patients with a substantial proportion of acral and mucosal melanoma, providing a preliminary framework for analyzing the patterns and timing of disease recurrence, which is an important step in developing surveillance strategies and adjuvant therapy schedule. Limited by the retrospective design and the lack of routine sentinel lymph node biopsies, further prospective studies are needed to confirm our conclusion.

## Data Availability

The datasets used or analyzed during the current study are available from the corresponding author on reasonable request.

## Abbreviations

CSD: Chronic sun-induced damage AJCC American Joint Committee on Cancer RFS Recurrence-free survival NCCN The National Comprehensive Cancer Network
